# Thermo-Responsive Shape Memory Effect and Conversion of Porous Structure in a Polyvinyl Chloride Foam

**DOI:** 10.3390/polym12092025

**Published:** 2020-09-04

**Authors:** Tao Xi Wang, Lu Lu Chang, Yun Hui Geng, Xing Shen

**Affiliations:** State Key Laboratory of Mechanics and Control of Mechanical Structures, Nanjing University of Aeronautics and Astronautics, 29 Yudao Street, Nanjing 210016, China; wa0003xi@nuaa.edu.cn (T.X.W.); luluchang@nuaa.edu.cn (L.L.C.); yhgeng@nuaa.edu.cn (Y.H.G.)

**Keywords:** shape memory effect, polyvinyl chloride, thermoset foam, porous structure

## Abstract

In this paper, a thermo-responsive shape memory effect in a polyvinyl chloride thermoset foam is characterized. Excellent shape recovery performance is observed in foam samples programmed both at room temperature and above their transition temperature. The conversion of porous structures in the foam from closed-cell to open-cell after a shape memory effect cycle is revealed via a series of specially designed oil-dripping experiments and optical images of the micro pores. Programming the strain higher than 20% results in an apparent increase in open-cell level, whereas programming temperatures have almost no influence.

## 1. Introduction

Purely synthetic thermoset polymeric foam has a wide range of applications in daily lives due to its light weight and tailorable mechanical properties [1,2]. In terms of cellular types, foam materials can be divided into two groups: closed-cell foam and open-cell foam. The difference between these two kinds of porous structures is how the air particles are entrapped within the solid. In closed-cell foams, cells are completely enclosed between each other by thin walls or membranes so that the air/liquid particles are restricted to that particular space inside the cell and are not able to move around from one cell to another. Cells are interconnected in open-cell foams, allowing liquid/air particles to pass through and move around between cells within the foam [3]. Compared to closed-cell foam, open-cell foam is light-weight and has low thermal conductivity, high soundproofing, and good water/air absorption and penetration. For these reasons, open-cell foam has a number of applications, such as acoustical engineering [4], footwear [5], packaging [6], etc.

Distinct differences can be found in the fabrication of these two types of foam. The manufacturing process of closed-cell foam is simpler. Casting/molding is used to create 3D shapes specific to the design and decomposition of foaming agents to produce clear pores [3,7]. For open-cell foams, the traditional vapor deposition [8] provides better control over the pore size, distribution, and interconnectivity. Compared with thermoplastic polymers that can rebuild their physical shapes and structures upon melting and solidification, most foams are thermoset so that their porous structures are completely fixed once fabricated. Switching between closed-cell and open-cell structures is traditionally not feasible. However, in this paper, we report a new finding that could realize structural conversion from closed-cell to open-cell in a commercial rigid polyvinyl chloride (PVC) foam using its shape memory effect (SME).

SME refers to the capability of a severely or quasi-plastically deformed material to recover its original shape in the presence of a certain stimulus [9,10,11]. Materials that undergo the SME are called shape memory materials (SMMs). A complete SME cycle includes two processes: programming, during which the SMM is deformed to a required/desired shape, and shape recovery, which can be either free (no external constraint) or constrained [12,13]. The external stimulus required to trigger the shape recovery can be heat (thermo-responsive SME) [14,15,16], chemicals (chemo-responsive SME) [10,17,18,19,20,21], light (photo-responsive SME) [22,23], or even magnetic fields (magneto-responsive SME) [24,25].

SMMs can be further categorized into two major groups: shape memory alloy (SMA) [25,26] and shape memory polymer (SMP) [27,28,29]. In comparison with SMAs, SMPs usually have much higher recoverable strain, easily tailorable thermo-mechanical properties, and better biocompatibility [30,31,32]. For these reasons, SMPs have attracted considerable attention in the past decade. So far, SMEs in both thermoset and thermoplastic polymers have been reported in the literature and many engineering polymers have been shown to have intrinsically superior shape memory performance [10,33,34,35,36,37,38]. In the case of foam materials, many foamed polymers (such as polyurethane (PU) [39], epoxy [40], ethylene-vinyl acetate copolymer (EVA) [41], etc.) have been reported with excellent SME when compression is used in programming.

In this work, the SME of the selected PVC foam was systematically characterized. Additionally, the conversion of its porous structure after an entire SME cycle was revealed via a series of specially designed oil-dripping experiments.

## 2. Materials, Sample Preparation, and Experimental Methods

**Figure 1:**
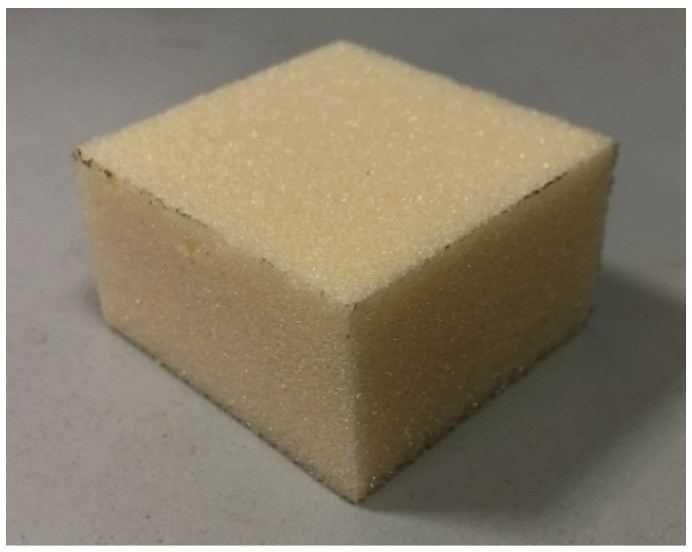
The polyvinyl chloride (PVC) foam used in this study.

As a combination of high mechanical strength and low density, the Divinycell H series PVC closed-cell thermoset foam from Diab Group, Sweden was used widely as the core material in sandwich composite structures. Divinycell H35 with 38 kg/m^3^ density was used in this study. Figure 1 depicts this foam.

**Figure 2:**
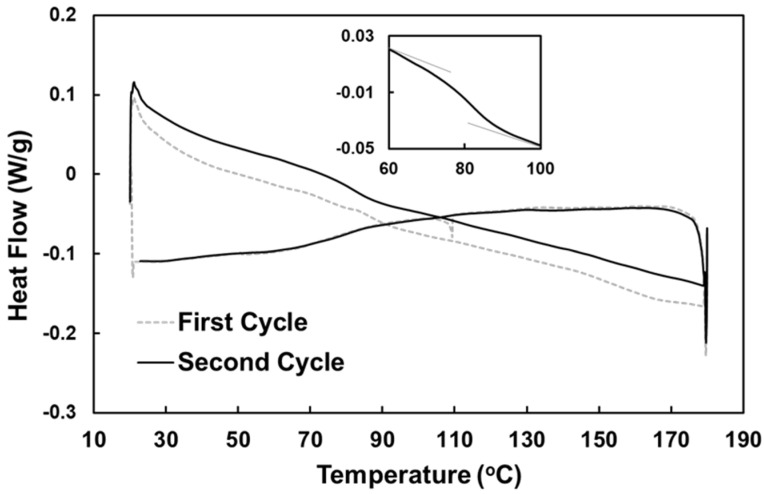
DSC results of the PVC foam. Inset: zoom-in view of the glass transition range during heating.

Figure 2 shows the differential scanning calorimetry (DSC) results of this foam, which were obtained from a Q200 DSC machine (TA Instruments, New Castle, DE, USA) under nitrogen flow at a heating/cooling speed of 10 °C/min for two thermal cycles to determine its thermal property.

The purpose of the first cycle was to eliminate the thermal history of the testing sample; thus, only the second cycle was used for analysis. As the figure shows, no clear melting/crystallization transition (peak and trough) was observed. A zoom-in view of the curve (inset of Figure 2) shows a step between 70 and 90 °C upon heating, which indicates the temperature range of glass transition (*T_g_*).

**Figure 3:**
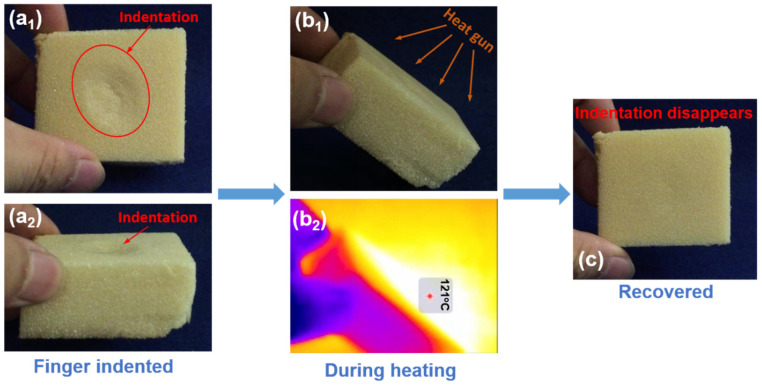
Demonstration of SME in the PVC foam. (**a_1_**) Top view of the indented foam sample; (**a_2_**) side view; (**b_1_**) foam sample during heating; (**b_2_**) infrared image of sample during heating; (**c**) top view of the recovered foam sample.

A simple demonstration of the thermo-responsive SME of the foam is shown in Figure 3. The cubic-shaped sample was initially finger-indented at room temperature (23 °C) on the central area of its upper surface. In Figure 3a_1_,a_2_, clear indentation can be seen in both top and side views. A heat gun was then used to heat the indented surface (Figure 3b_1_). In the infrared photo (Figure 3b_2_), the highest surface temperature is about 121 °C, which is well above its *T_g_*. During heating, gradual flattening was observed on the indented area. After heating for around 30 s, the indentation completely disappeared and the sample recovered its original flat surface.

In this study, the investigation of the PVC foam had two parts. The first part focused on the characterization of its SME, whereas the second part introduced the SME-based structural conversion from closed-cell to open-cell.

**Figure 4:**
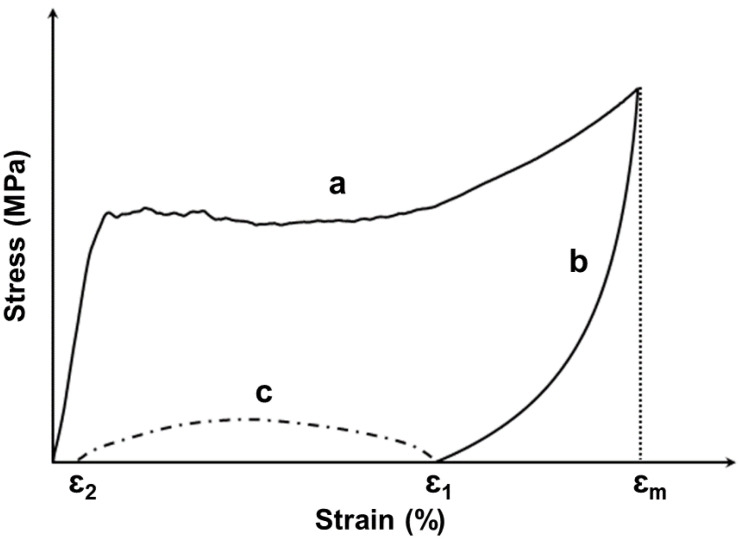
Illustration of a typical shape memory cycle.

In the first part, cubic shaped samples were prepared with dimensions of 25 mm (length) × 25 mm (width) × 30 mm (thickness). As illustrated in Figure 4, a complete SME cycle contained two processes and three steps in this study:

(a) At room temperature or 80 °C, the sample was uniaxially compressed to a required maximum strain (*ε_m_*) of 20%, 50%, or 80% at a constant strain rate of 10^−2^/s.

(b) After cooling back to room temperature (if necessary), the load was gradually released at the same strain rate. The residual strain after unloading is denoted as *ε*_1_. This represents the end of programming process.

(c) Samples were reheated to 100 °C (above *T_g_*) for 10 min for recovery. The residual strain after recovery is denoted as *ε*_2_. This is the shape recovery process. Note that a 100% shape recovery could be achieved if *ε*_2_ = 0.

Thus, we can define the shape fixity ratio (*R_f_*) to evaluate the capability of the material to maintain the deformed shape,

(1) $$R_{f}=\varepsilon_{1}/\varepsilon_{m}$$

and the shape recovery ratio (*R_r_*) for the capability of the material to restore its original shape,

(2) $$R_{r}=\left(\varepsilon_{1}-\varepsilon_{2}\right)/\varepsilon_{1}.$$

Here, unless otherwise stated, the compression/programming was conducted using an Instron 5565 machine with an integrated temperature-controlled chamber. For simplicity, all strain and stress mentioned are meant for engineering strain and engineering stress.

In addition, a conical indenter with a 120° cone angle and a spherical indenter with a diameter of 1.588 mm were used to produce local indentation on foam surfaces. The sample was then heated at 100 °C for 5 min to induce local shape recovery. The surface topography of these two samples was recorded after both indentation and recovery using a Sensofar S neox 3D optical profilometer.

In the second part, a series of oil-dripping experiments were carried out in the foam. Cubic foam samples that were 4 mm thick with a width and length of 20 mm were prepared and initially programmed to *ε_m_* values of 20%, 50%, and 80% at either room temperature or 80 °C. After heating for shape recovery, two drops of silicone oil were then dripped onto the upper surfaces of each sample. An original foam sample (without treatment) with the same size was also used as reference. A piece of color paper was placed underneath each sample so that clear staining could be observed once the oil flowed through the sample and reached the bottom. The staining results were captured by camera after 4, 8, 14, and 24 h upon dripping. With the help of the software Adobe Photoshop, the open-cell levels of these samples were quantified using the nominal stained area *S*, which is defined by:

(3) $$S=N_{d}/N_{p}$$

where *N_d_* and *N_p_* represent the pixel number of dripped area and the entire paper, respectively.

Optical images of the foam samples both before and after structural conversion were taken using a microscope. The foam samples that recovered from *ε_m_* of 80% were compressed to 80% once again at room temperature to reveal the mechanical strength of the foam after structural conversion.

## 3. Experimental Results and Analysis

### 3.1. Characterization of Shape Memory Effect (SME)

**Figure 5:**
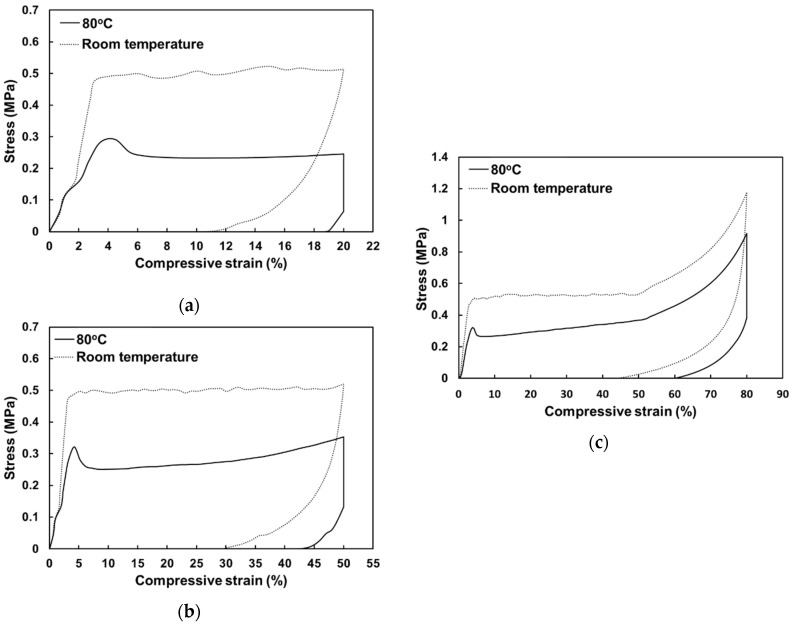
Typical stress vs. strain relationships in uniaxial compression to a maximum strain of 20% (**a**), 50% (**b**), and 80% (**c**) at room temperature and 80 °C (followed by cooling back to room temperature) and then unloading.

The typical stress versus strain relationship of the foam under uniaxial compression to a maximum strain of 20% at both room temperature and 80 °C are plotted in Figure 5a. The figure shows that the residual strain for the sample programmed at room temperature was 10.88%, which was much higher (18.68%) for the sample programmed at 80 °C. In terms of stress, the two samples behaved almost the same before a strain of 1%. However, from 2% onwards, the stress of the sample programmed at 80 °C became much lower and the stress after strain of 6% was about half of the stress of the one programmed at room temperature. The difference in stress resulted from the glass transition induced softening.

A similar trend was observed in samples with *ε_m_* of 50% in Figure 5b. After strain of around 30%, slight hardening occurred in the sample at 80 °C, whereas it maintained a rather stable stress level at room temperature. After unloading, the sample programmed at 80 °C maintained a much higher residual strain compared with the one programmed at room temperature (42.00% vs. 29.25%).

Figure 5c presents the results of samples with an *ε_m_* of 80%, confirming that high testing temperature causes lower compressive stress but better shape fixing. In addition, severe stress hardening was observed in both samples when compression was more than 50%.

**Figure 6:**
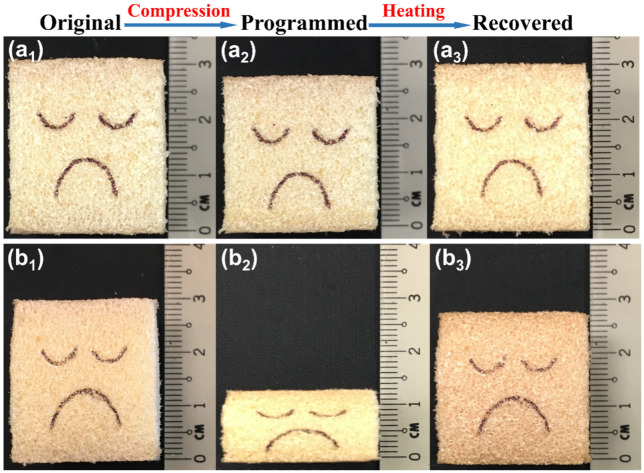
Evolution of thickness of the foam. (**a_1_**) Original sample; (**a_2_**) sample programmed to *ε_m_* of 20% at room temperature; (**a_3_**) recovered sample; (**b_1_**) original sample; (**b_2_**) sample programmed to *ε_m_* of 80% at 80 °C; (**b_3_**) recovered sample.

The evolution in thickness of two typical samples after heating for shape recovery is presented in Figure 6; Figure 6a,b represent the samples programmed to 20% at room temperature and 80% at 80 °C, respectively.

As we can see from (a_3_), the sample seemingly returned to its original thickness upon heating for recovery. However, a tiny residual strain *ε*_2_ (1.83%) was determined after precise measurement by a digital Vernier caliper. For the sample in (b), residual strain *ε*_2_ was 6.64% after recovery. With the exception of these two samples, the *ε*_2_ of tested samples was also recorded and the results are summarized together with *ε*_1_ in Table 1 for further investigation.

**Figure 7:**
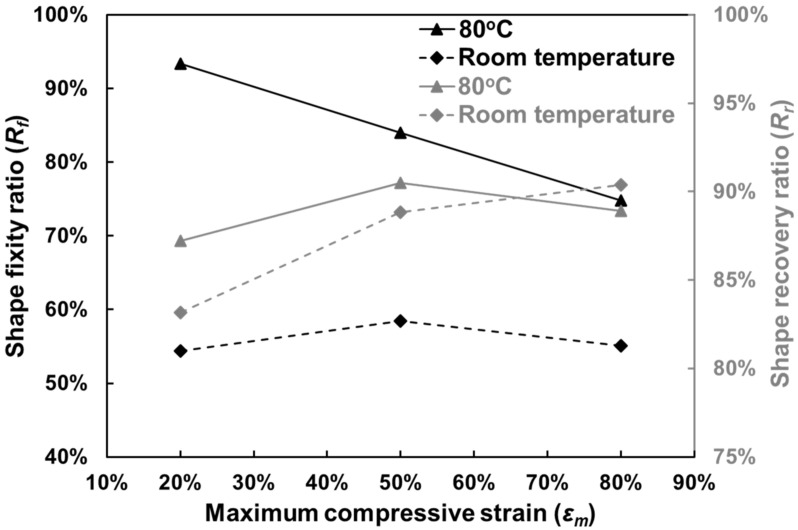
Shape fixity ratios (black color) and shape recovery ratios (grey color) of all tested samples as a function of *ε_m_*.

With *ε*_1_ and *ε*_2_, the shape fixity ratio (*R_f_*) and shape recovery ratio (*R_r_*) were then obtained and the results are presented in Figure 7.

Clearly, programming at 80 °C causes much higher *R_f_* regardless of the deformation level. However, *R_f_* at 80 °C keeps decreasing in nearly linearly with the increase in maximum strain. The highest *R_f_* was 93.4% occurring at an *ε_m_* of 20%, which dropped to only 74.8% at an *ε_m_* of 80%. The situation was quite different in room temperature programming as the corresponding *R_f_* (black dash-line) was almost deformation-independent (around 55%).

*R_r_* was higher than 80% in all samples. Unlike *R_f_*, not much difference was observed in *R_r_* between room temperature programming and 80 °C programming. Additionally, *R_r_* for 80 °C programming stayed high and stable (between 87% and 91%) regardless of *ε_m_*. Therefore, we conclude that the foam has quite outstanding shape recovery performance in both room temperature and 80 °C programming.

**Figure 8:**
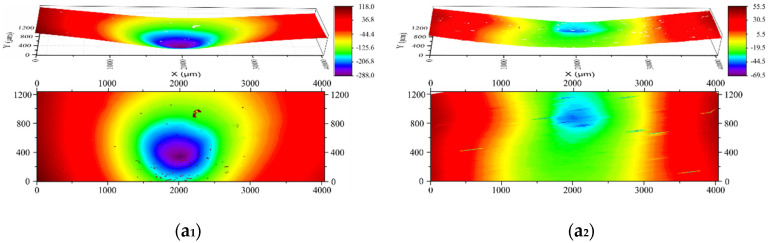

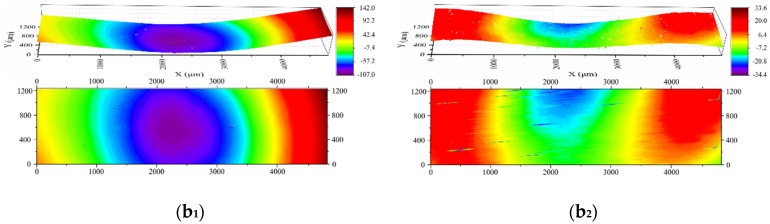
Evolution of surface topography of the indented foam samples. (**a_1_**) After indentation with a conical indenter; (**a_2_**) upon heating at 100 °C for 5 min; (**b_1_**) after indentation with a spherical indenter; (**b_2_**) upon heating at 100 °C for 5 min.

**Figure 9:**
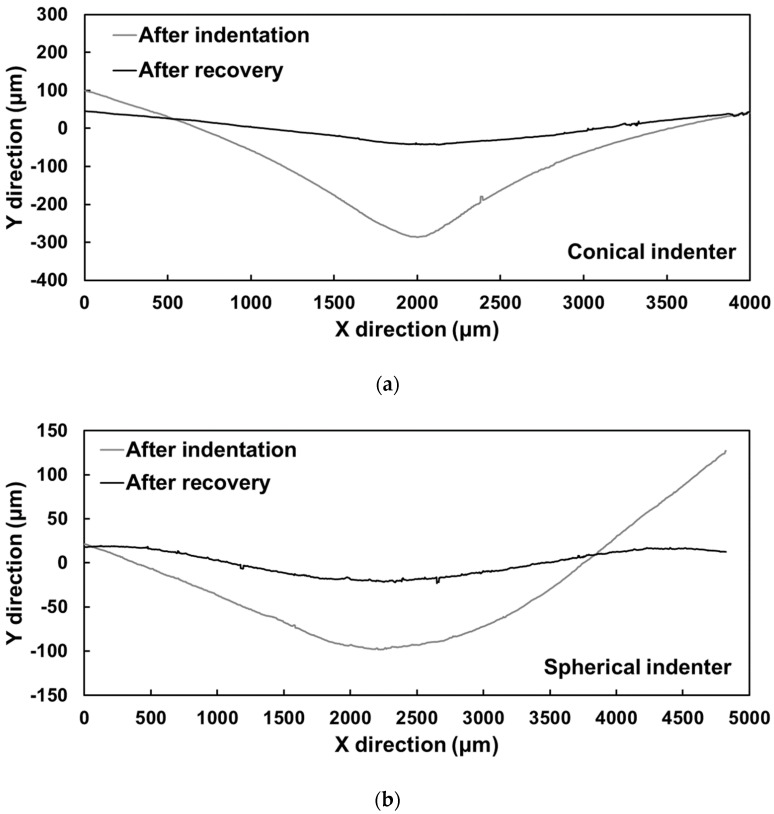
Comparison between cross-sections of foam samples after indentation (black solid lines) and recovery (grey solid lines). (**a**) Sample indented with conical indenter; (**b**) sample indented with spherical indenter.

The evolution of the surface topography of the indented foam samples is presented in Figure 8, where Figure 8a,b represent indentation by conical and spherical indenters, respectively. Subscripts 1 and 2 refer to the sample after indentation and recovery, respectively. As shown in Figure 8a_1_,b_1_, the maximum depths of conical and spherical indentation were 288 and 107 μm, respectively. After heating for recovery, the values reduced to 69.5 and 34.4 μm, which indicated a 76% and 68% recovery in depth, respectively. In terms of overall surface topography, although slight surface unevenness was still observable in both samples after recovery, the indentation disappeared. Figure 9 shows the 2D cross-sectional view of the indented surfaces, and clear surface flattening was also observed in both samples after recovery. 

### 3.2. Structural Conversion from Closed-Cell to Open-Cell

**Figure 10:**
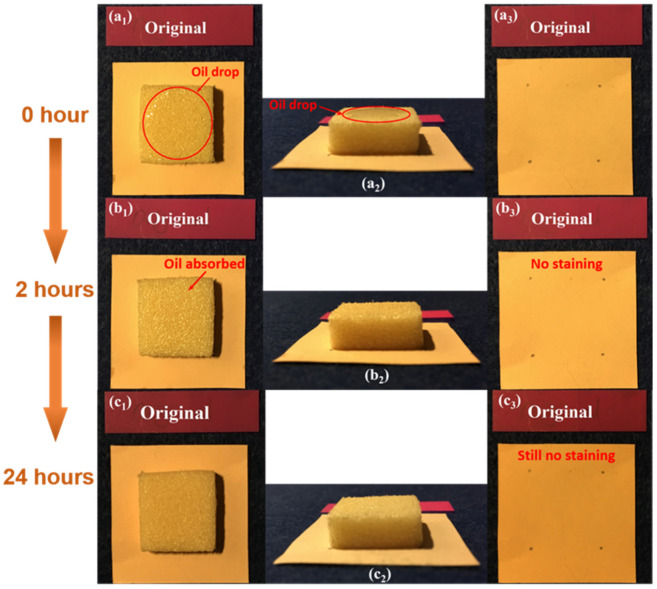
Oil-dripping experiment in an untreated foam sample. (**a_1_**) Top view of sample upon dripping; (**a_2_**) side view of sample upon dripping; (**a_3_**) top view of the paper underneath upon dripping; (**b_1_**) top view of sample after 2 h upon dripping; (**b_2_**) side view of sample after 2 h upon dripping; (**b_3_**) top view of the paper underneath after 2 h upon dripping; (**c_1_**) top view of sample after 24 h upon dripping; (**c_2_**) side view of sample after 24 h upon dripping; (**c_3_**) top view of the paper underneath after 24 h upon dripping.

The results of the oil-dripping experiment on the untreated foam sample are presented in Figure 10, in which Figure 10a–c were recorded right after dripping, after 2 h upon dripping, and after 24 h upon dripping, respectively. As revealed in Figure 10a_2_, oil drop was on the foam in a nearly flat circular shape due to its hydrophobic surface. Four grey reference dots on the paper underneath the foam were drawn to indicate the position of the foam. As shown in Figure 10b_1_,b_2_, oil was found to be fully absorbed into the foam after 2 h upon dripping. However, no staining was found on the paper (Figure 10b_3_). The same result was obtained after 24 h (Figure 10c_1_–c_3_). Therefore, we conclude that the oil was trapped inside the pores, indicating the closed-cell porous structure of the foam.

**Figure 11:**
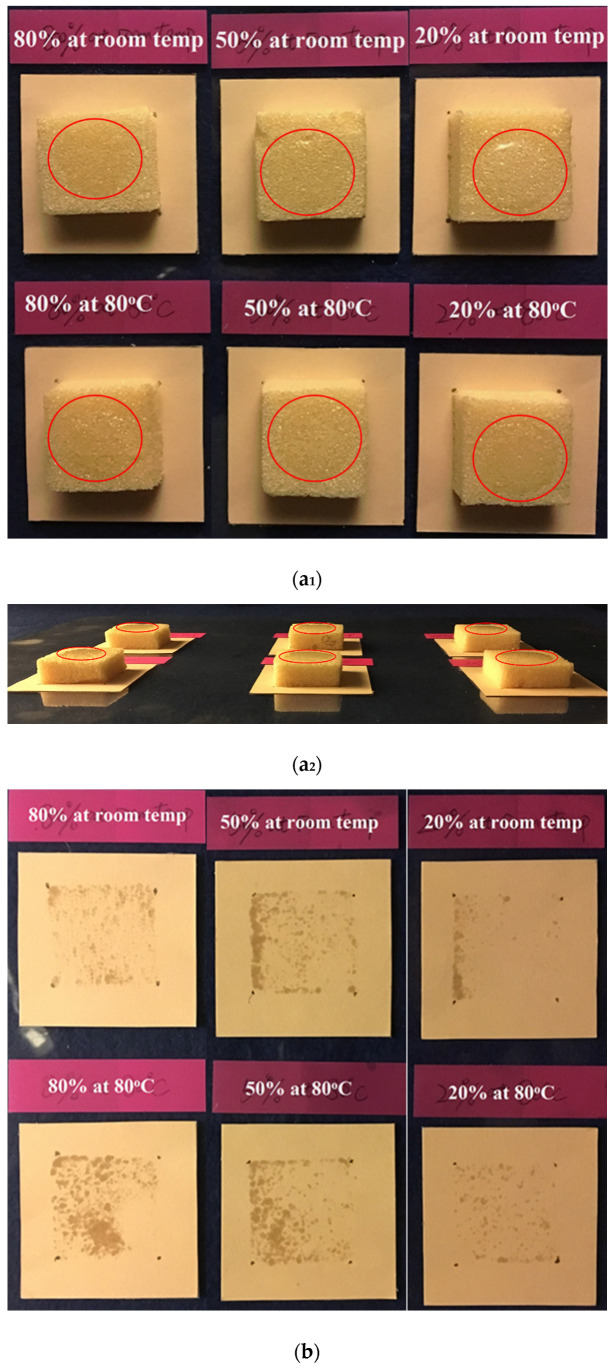

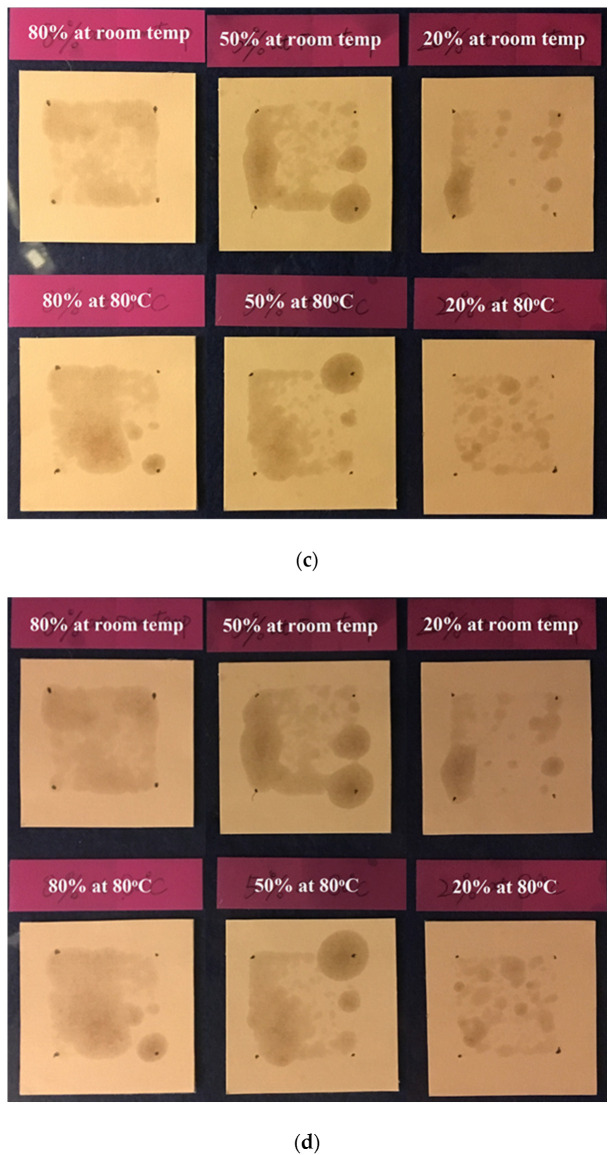

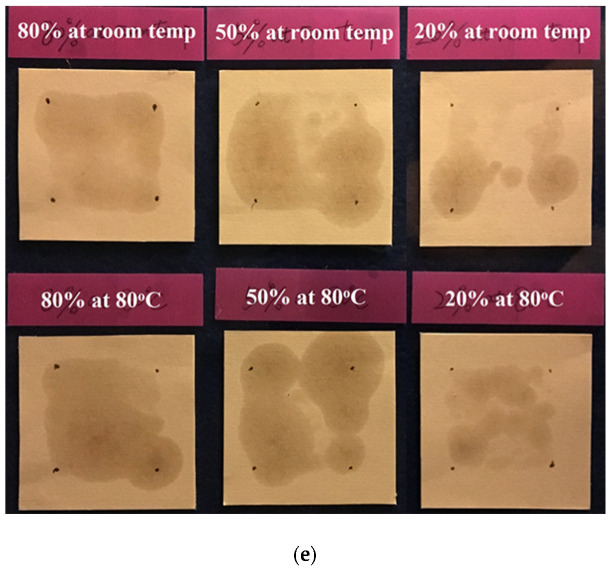
Oil-dripping experiments in foam samples after shape recovery from programming at room temperature (80 °C) to *ε_m_* of 20%, 50% and 80%. (**a_1_**) Samples upon dripping; (**a_2_**) side view upon dripping; (**b**–**e**) top views of the papers underneath after 4, 8, 14, and 24 h upon dripping, respectively. Red circles in (**a**) and (**a_1_**) indicate the oil drops.

The experimental results of the foam cubes after SME cycles are presented in Figure 11. The corresponding *ε_m_* and programming temperature were marked above each sample. Top and side views of all samples right after dripping are shown in Figure 11a_1_,a_2_, respectively. Red circles were drawn on the pictures to indicate the positions of oil drops.

Results after 4, 8, 14 and 24 h upon dripping are presented in (b), (c), (d) and (e), respectively. As shown, oil staining was found on each of them, which indicated the interconnection between pores so that oil could flow from one pore to another and eventually outflow the foam body. Therefore, open cells were created inside the foam after a SME cycle.

**Figure 12:**
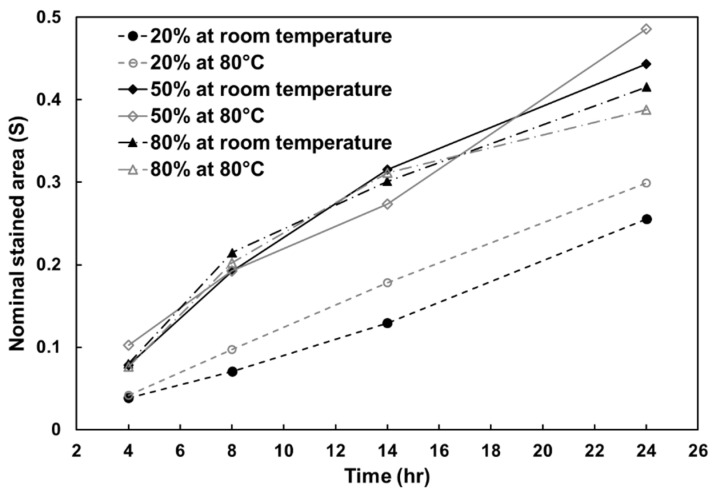
Nominal stained area vs. time (in hours) upon dripping.

The relationship between nominal staining area (*S*) and time of all samples is plotted in Figure 12. Samples with an *ε_m_* of 20% had the lowest open-cell level and their *S* grew almost linearly (dashed lines), regardless of programming temperature. The open-cell level in samples with an *ε_m_* of 50% and 80% were quite similar (solid and dash-dot lines) but apparently higher in the one with *ε_m_* of 20%. This phenomenon is also evidenced in Figure 11e, where almost the entire contact area on papers for samples with *ε_m_* of 50% and 80% were stained after 24 h, whereas the unstained region was still obvious for those with *ε_m_* of 20%. However, programming temperature seemingly has no apparent influence on open-cell level as the *S* values in samples with same *ε_m_* are all quite close.

**Figure 13:**
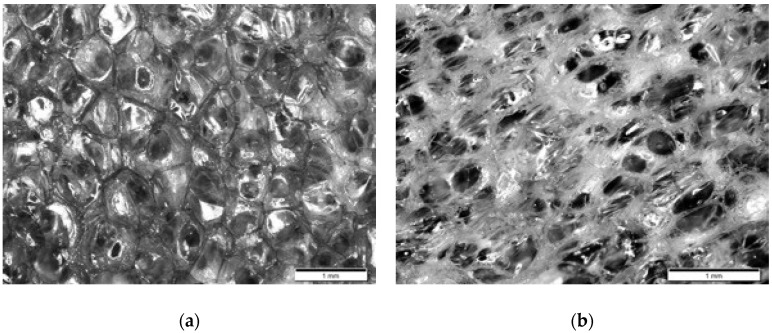
Optical images of the cross-section of the original foam (**a**) and the foam recovered from programming to 80% at room temperature (**b**). The scale bar is 1 mm.

Optical images of the cross-section of the foam are presented in Figure 13, in which Figure 13a,b refer to the original foam and the foam recovered from programming with *ε_m_* of 80% at room temperature, respectively. As shown in Figure 13a, thin membranes that are highly reflective to lights were seen within each cell. The membranes separate pores so that air/liquid could only be trapped inside instead of flowing around. The recovered foam sample in Figure 13b is different. The reflective membranes seemingly disappeared, and the pores looked much clearer in the image, which visually confirmed the interconnection of the pores after a SME cycle. With such structural conversion, flowing of air/liquid inside the foam body becomes possible, resulting in the staining phenomenon observed in Figure 11.

**Figure 14:**
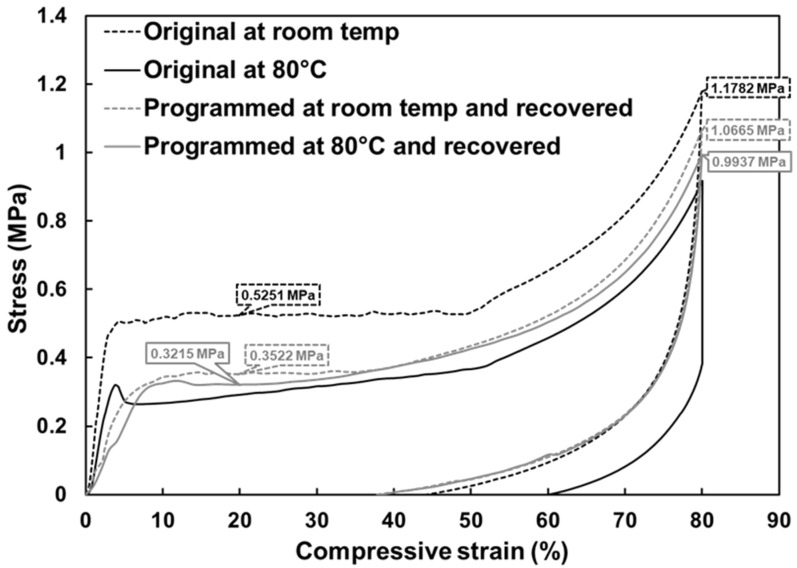
Comparison of stress and strain relationships under uniaxial compression to 80% strain between original samples at room temperature and 80 °C, and shape-recovered samples with *ε_m_* of 80% at room temperature.

Figure 14 compares the mechanical behaviors of the original foam and shape-recovered foam under uniaxial compression to 80%. Note that for the shape-recovered samples, the *ε_m_* was 80% and programming at both room temperature and 80 °C are included (grey dashed and solid lines). Results show only a slight difference between the recovered samples, which indicates that programming temperatures have almost no influence on the mechanical strength of the foam. On the other hand, the stress of these two samples at strain of 20% were 0.3522 and 0.3215 MPa, respectively, which are 67.0% and 61.2% of that of the original sample at room temperature (0.5251 MPa). In terms of the maximum stresses (at 80% strain), the ratios were approximately 90.5% and 84.3%, respectively. Additionally, compared to the original sample at 80 °C, shape-recovered samples had slightly higher stress during loading. The results indicate that despite the porous structure being converted to open-cell, the mechanical strength of the foam after a SME cycle is still considerable.

## 4. Discussion

The oil-dripping experiment and optical images of the cross-section prove the closed-cell to open-cell conversion of the porous structure of such foam. A potential interpretation may be that the compression of the foam during programming squeezes the pores and destroys the thin membranes. Upon heating, the compressed pores recover their original sizes without membranes, which consequently creates many open cells.

Although the foam used in this study was PVC-based, such structural conversion may not be limited to this material. This technique can be considered as a potential general technique to produce open-cell structure even for other types of polymeric foam, since SME is an intrinsic property of many engineering polymers [11]. From a manufacturing point of view, the fabrication of rigid open-cell polymeric foam may also be simplified by such structural conversion because producing foam with closed-cell structures is currently much easier in foam industry.

H35 PVC foam is widely used as the core material of industrial composite sandwich structures; thus, deformation caused by external load is likely to happen in real applications. Deformed structures lose some of their mechanical rigidity and have a higher risk of causing accidents, and thus are considered unsuitable for carrying on work. However, replacing local deformed parts is impractical. With the experimental results of this study, a potential solution based on SME may have emerged. From Figure 7 and Figure 14, the foam is able to not only recover the sizes via heating, but also restore most of its mechanical strength, so that reusing the deformed foam becomes possible. Moreover, re-process thermosets remains challenging, so reusing the foam could consequently reduce the concern of recycling as well.

## 5. Conclusions

In this paper, a systematic characterization of the SME in a commercial PVC foam was presented. After programming at either 80 °C or room temperature, excellent shape recovery was always observed upon heating. The programming destroyed the membranes between pores so that after shape recovery, the original closed-cell porous structure of the foam converted to open-cell. An apparent increase of the open-cell level is confirmed in foam with *ε_m_* higher than 20%. In addition, the mechanical strength was also revealed to be largely maintained after structural conversion. However, programming temperatures seemingly have almost no influence on both the open-cell level and mechanical strength of the foam. Such structural conversion may also be extended to other types of polymeric foam as a new general technique to fabricate open-cell structures. The capabilities of both shape and strength recovery may allow the deformed foam to be reused in real applications.

## Figures and Tables

**Table 1:** *ε*_1_ and *ε*_2_ of all tested foam samples.

	20%		50%		80%	
	*ε*_1_	*ε*_2_	*ε*_1_	*ε*_2_	*ε*_1_	*ε*_2_
**Room temperature**	10.88%	1.83%	29.95%	3.27%	44.05%	4.24%
**80 °C**	18.68%	2.39%	42.00%	3.98%	59.84%	6.64%
